# Chromosome-level genome assembly of milk thistle (*Silybum marianum* (L.) Gaertn.)

**DOI:** 10.1038/s41597-024-03178-3

**Published:** 2024-04-05

**Authors:** Kyung Do Kim, Jeehyoung Shim, Ji-Hun Hwang, Daegwan Kim, Moaine El Baidouri, Soyeon Park, Jiyong Song, Yeisoo Yu, Keunpyo Lee, Byoung-Ohg Ahn, Su Young Hong, Joong Hyoun Chin

**Affiliations:** 1 Department of Biosciences and Bioinformatics, Myongji University, Yongin, 17058 Korea; 2 EL&I Co., Ltd., Hwaseong, 18278 Korea; 3 Department of Research and Development, DNACARE Co. Ltd., Seoul, 06126 Korea; 4 Laboratoire Génome et Développement des Plantes, Center National de la Recherche Scientifique (CNRS), Perpignan, France; 5 Laboratoire Génome et Développement des Plantes, University of Perpignan Via Domitia, Perpignan, France; 6 International Technology Cooperation Center, Technology Cooperation Bureau, Rural Development Administration, Jeonju, 54875 Korea; 7 Genomics Division, Department of Agricultural Biotechnology, National Institute of Agricultural Science, Rural Development Administration, Jeonju, 54874 Korea; 8 Food Crops Molecular Breeding Laboratory, Department of Integrative Biological Sciences and Industry, Sejong University, Seoul, 05006 Korea; 9 Convergence Research Center for Natural Products, Sejong University, Seoul, 05006 Korea

**Keywords:** Comparative genomics, Plant evolution

## Abstract

*Silybum marianum* (L.) Gaertn., commonly known as milk thistle, is a medicinal plant belonging to the Asteraceae family. This plant has been recognized for its medicinal properties for over 2,000 years. However, the genome of this plant remains largely undiscovered, having no reference genome at a chromosomal level. Here, we assembled the chromosome-level genome of *S. marianum*, allowing for the annotation of 53,552 genes and the identification of transposable elements comprising 58% of the genome. The genome assembly from this study showed 99.1% completeness as determined by BUSCO assessment, while the previous assembly (ASM154182v1) showed 36.7%. Functional annotation of the predicted genes showed 50,329 genes (94% of total genes) with known protein functions in public databases. Comparative genome analysis among Asteraceae plants revealed a striking conservation of collinearity between *S. marianum* and *C. cardunculus*. The genomic information generated from this study will be a valuable resource for milk thistle breeding and for use by the larger research community.

## Background & Summary

*Silybum marianum* (L.) Gaertn., commonly known as milk thistle, is an annual or biennial plant belonging to the *Asteraceae* family^1–3^ and has been recognized for its medicinal properties for over 2,000 years^4,5^. Silymarin, a complex of flavonolignans extracted from milk thistle seeds^6–8^, exhibits remarkable hepatoprotective and detoxifying effects^9–14^. In recent years, it has garnered attention as a potential therapeutic agent for various liver ailments, including alcoholic liver disease and acute viral hepatitis^15–19^.

Despite being a distinct species from *Cirsium* spp., milk thistle is often misidentified due to phenotypic similarities. Therefore, deciphering the milk thistle genome holds immense value in understanding and optimizing the plant’s beneficial properties. Sequencing the milk thistle genome can help researchers understand the molecular mechanisms underlying silymarin’s therapeutic properties and identify new compounds with potential medicinal applications. Additionally, it can help identify genes that can be manipulated to increase silymarin production. This knowledge can also help develop strategies to protect plants from pests and diseases. Despite the growing recognition of silymarin’s therapeutic potential, the genome of *S. marianum* remains largely uncharted. Yet, there is no reference genome sequenced for *S. marianum* at the chromosomal level. This lack of genomic resources poses a significant hurdle to advancing research and plant breeding on *S. marianum*.

To bridge this gap, we assembled the chromosome-level genome of *S. marianum* using a combination of Oxford Nanopore long-read, Illumina short-read, and Pore-C technologies. This study unveiled the genetic landscape and diversity of the plant, allowing for the annotation of 53,552 genes and the identification of transposable elements consisting of 58% of the genome. The genomic resources, gene structure, and functional insights generated from this study will pave the way for future research efforts aimed at harnessing the full potential of milk thistle.

## Methods

### Sample preparation and genomic sequencing

**Fig. 1:**
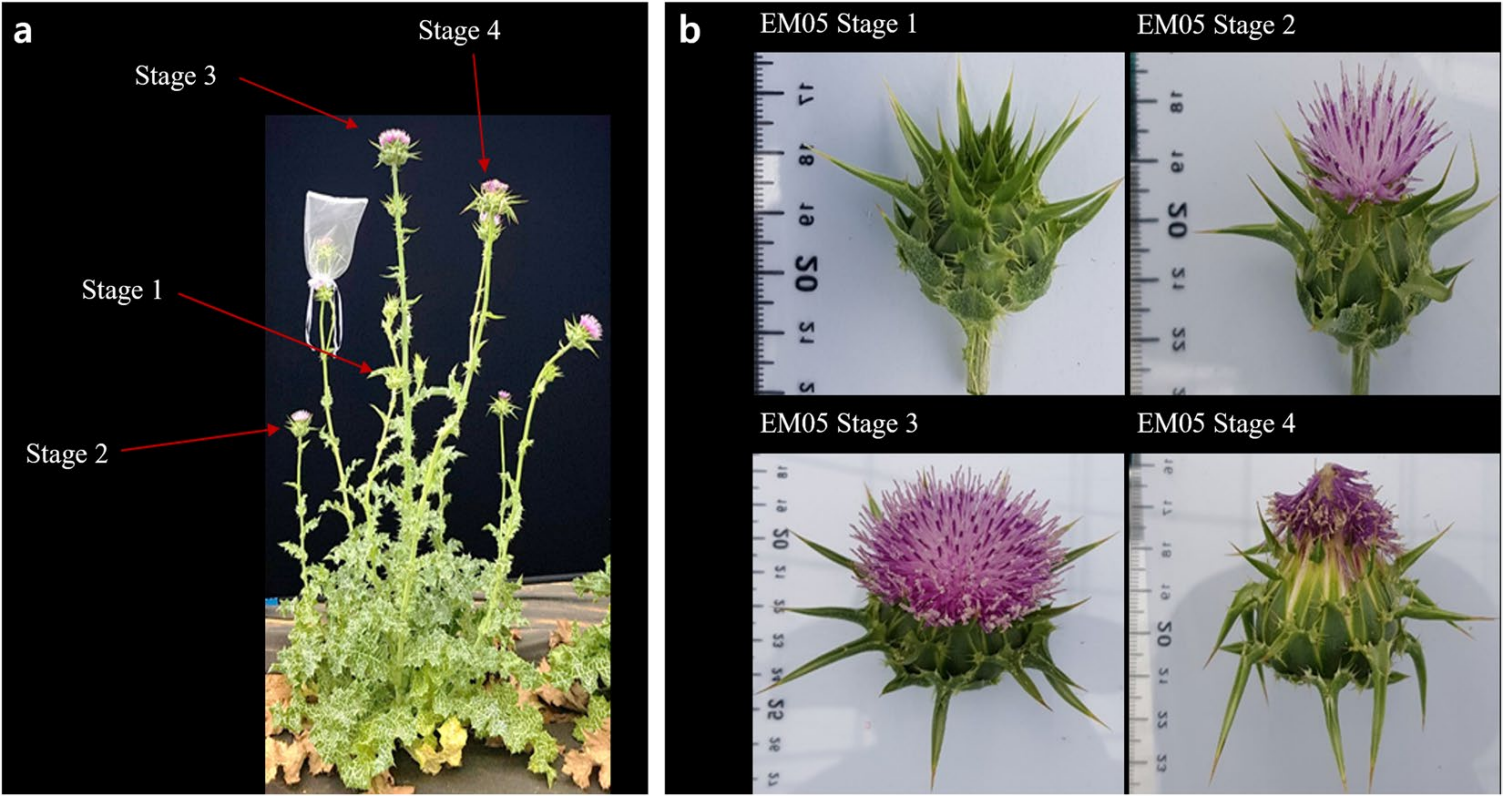
Morphology and flowering stages of *Silybum marianum*. (**a**) Morphology of *S. marianum* plant. Each arrow reflects different flowering stages. (**b**) Flowering stages of *S. marianum*. Stage 1: No petals have emerged, and small white seeds are visible near the base of the flower receptacle. Stage 2: Some petals have emerged, and small white seeds are visible near the base of the flower receptacle. Stage 3: Most petals have emerged, but they are not yet withered. Slightly larger white seeds are visible near the flower receptacle. Stage 4: Most petals have emerged, but they are withered. The flower receptacle has thickened, and the seeds are larger and firmer.

*Silybum marianum* cv. ‘Silyking’, also known as ‘EM05’, is a patented variety recognized for its abundant silymarin content (Fig. 1a). EM05 originated from germplasm collected in 2017 at local farms in Pyeongtaek, Gyeonggi-do, Korea. It was carefully selected from heterogeneously collected accession and self-propagated to achieve the pure line of ‘EM05’ by EL&I Co., ltd. in Hwaseong, Gyeonggi-do, Korea. Genomic DNA was extracted from young leaves of EM05 using the Cetyltrimethylammonium Bromide (CTAB) method. The quality and quantity of the extracted DNA were assessed using NanoDrop 2000 (Thermo Fisher Scientific, USA).

Nanopore library was prepared using a ligation sequencing kit, SQK-LSK110. Long-read sequencing was performed using an FLO-PRO002 flow cell on the Oxford Nanopore PromethlON platform. A total of 77.31 Gb raw data with an average read length of 25.24 Kb and an N50 length of 39.84 Kb were obtained, accounting for ~ 111.3-folds of the genome (Table 1). Illumina paired-end library with a 400 bp insert size was prepared using the TruSeq Nano DNA kit. Short-read sequencing was conducted on the Novaseq 6000 platform with 2 × 150 bp reads, which generated 52.24 Gb raw data, accounting for ~ 75.23-folds of the genome (Table 1). The low-quality sequences with a Phred score of 20 or lower, as well as Illumina adapter sequences, were removed using Trimmomatic v.0.39^20^.

**Table 1:** Comparison of genome assemblies between *Silybum marianum* ASM154182v1 and cv. Silyking v1.

		*S. marianum* ASM154182v1	*S. marianum* cv. Silyking v1 (this study)
**Long-read sequencing platform**		PacBio	Oxford Nanopore
**Genome coverage**	Long-read (fold)	—	111.3
	Short-read (fold)	—	75.23
	Hi-C (bp; Total/N50)	—	39.5 Gb/5.4 kb
**Estimated genome size (Mb)**		1,477.58	694.4
**Estimated heterozygosity (%)**		—	0.172
**Number of scaffolds**		—	17
**Total length of scaffolds (Mb)**		—	689.3
**Scaffold N50 (Mb)**		—	41.4
**Longest scaffold (Mb)**		—	60.7
**Number of contigs**		258,575	70
**Total length of contigs (Mb)**		1,477.57	5.1
**Contig N50 (Mb)**		0.006967	0.25
**Contig L50**		62,112	9
**Longest contig (Mb)**		0.099455	0.39
**GC content (%)**		37.2	34.5
**Mapping rate of Illumina reads (%)**		—	99.4
**Completeness BUSCO (%)**		36.7	99.1
**Completeness single-copy BUSCO (%)**		31.7	95.1
**Completeness duplicated BUSCO (%)**		5.0	4.0

### Transcriptome sequencing

For RNA extraction, seven tissue samples from various parts of the *S. marianum* EM05 plant, including flowers, leaves, stems, and roots, were collected. For flower tissues, samples from four different stages of inflorescence were collected (Fig. 1b).

Total RNA was extracted using CTAB buffer (OPS Diagnostics, USA) with the addition of 50 µL of β-Mercaptoethanol to 500 ml of the buffer. The process involved mixing the samples with 900 µL of CTAB buffer, followed by centrifugation at 14000 rpm for 5 minutes at 4 °C. The resulting 1st supernatant (700 µL) was then incubated at 65 °C for 15 minutes with intermittent vortexing. The lysate was mixed with an equal volume of Phenol: chloroform: isoamyl alcohol (25:24:1) PCI and centrifuged at 14000 rpm for 5 minutes at 4 °C. Subsequently, the 600 µL supernatant was mixed with LiCl(5 M) in a 1:1 ratio, incubated at −20 °C for 4 hours, and then centrifuged at 14000 rpm for 10 minutes at 4 °C. After removing the supernatant, a 500 µL wash with 70% Ethanol was performed, followed by centrifugation at 14000 rpm for 3 minutes at 4 °C. The samples were air-dried for 20 minutes before adding 50 µL elution buffer (0.1x TX buffer) with thorough mixing. For DNase1 treatment, QIAGEN DNase1 powder was dissolved in 550 µL H2O and then aliquoted into 1.5 ml E-Tube in each tube. Just before use, buffer was added to DNase1 in a 1:1 ratio. Incubation was carried out at 37 °C for 30 minutes.

RNA sequencing library was prepared using TruSeq Stranded mRNA Sample Preparation Kit and sequenced on the Novaseq 6000 platform with 2 × 101 bp reads. A total of 36 Gb of raw data with an average of 52 million reads per sample was generated from seven *S. marianum* samples.

### Genome assembly and chromosome-level scaffolding

The characteristics of the *S. marianum* genome were estimated based on a total of 304,981,656 trimmed Illumina read pairs with 151 bp in length. The distribution of *k*-mer read depth was computed using Jellyfish v2.2.10^21^, and the genome size and heterozygosity were calculated using GenomeScope v2.0^22^ with default parameters. In this study, *k*-mer values of 19 and 21 were used. The estimated genome size was 643 Mb with 0.151% heterozygosity using 19-mer and 654 Mb with 0.146% heterozygosity using 21-mer (Table 1, Figure S1).

The draft genome of *S. marianum* was assembled using Oxford Nanopore long-reads with Nextdenovo v2.5.0^23^. The assembly resulted in 70 contigs with a total length of 706 Mbp. Gap sequences in the draft genome were polished using Illumina short-reads with NextPolish v1.4.0^24^.

**Fig. 2:**
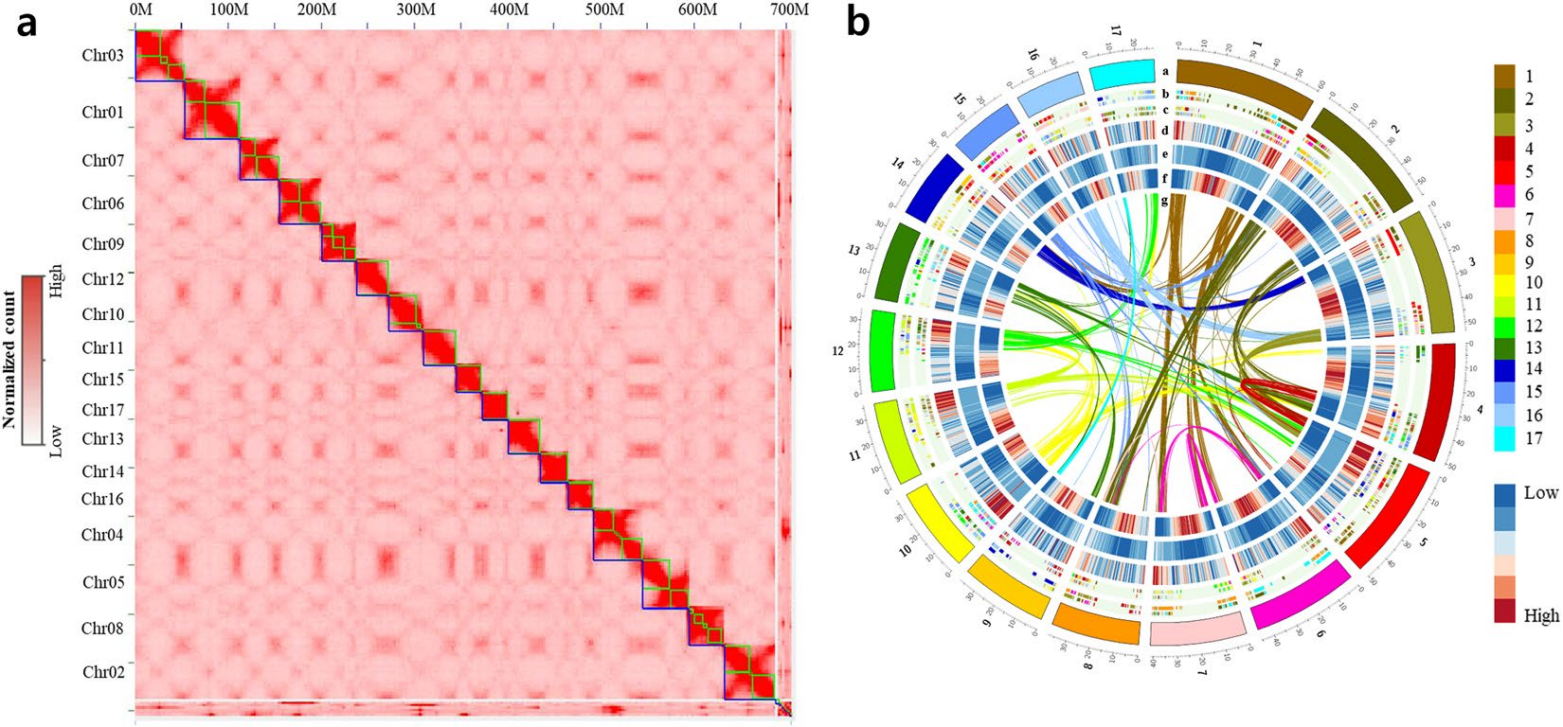
Overview of the genomic landscape of *Silybum marianum*. (**a**) Pore-C interaction heatmap of *S. marianum* assembly. The interactions of the *S. marianum* chromosome were measured by the number of the Pore-C reads illustrated by red color. (**b**) Genome features of *S. marianum* across the 17 chromosomes. Each track was drawn in a 500 kb window. The outer to the inner tracks represent: a. Chromosomes of *S. marianum*; b. Synteny regions between *Cynara cardunculus* and *S. marianum*; c. Synteny regions between *Helianthus annuus* and *S. marianum*; d. Gene count of *S. marianum* in 500 kb; e. DNA TE count of *S. marianum* in 500 kb; f. LTR TE count of *S. marianum* in 500 kb. g. Curved lines at the center show segmental duplication regions in *S. marianum*. Each color labeled at the track a, b, c, and g represents each chromosome.

To assemble the chromosome-level genome, a Pore-C library was prepared. This involved various steps such as nuclei isolation, chromatin denaturation, digestion, ligation, de-crosslinking, and DNA extraction. Library construction was carried out using the extracted DNA and the SQL-LSK110 ligation kit (Oxford Nanopore) following the manufacturer’s protocol. The constructed libraries were checked for quality on a 1.0% TBE agarose gel. The Pore-C library was sequenced using the Oxford Nanopore PromethION platform, generating 39.47 Gbp of raw data (Table 1). The raw data was trimmed using Guppy v3.0.4 with Q >  = 7, resulting in 34.05 Gbp of raw data with a mean quality of 11.9. Only the trimmed data was statistically assessed with anoPlot v1.40.0. Mapping of trimmed Pore-C data to the assembly and removal of duplicated alignments were performed using Pore-C Snakemake v0.4.0. Assembly, hic, and fastq files were created using 3D-DNA pipeline v180922. The assembly was manually curated based on the pairwise contact heatmap (Fig. 2a) generated using JuiceBox v1.11.08^25^. After scaffolding, a total of 35 contigs were connected into 17 chromosome-level scaffolds with a total length of 689.3 Mbp (Table S1). Unplaced contigs showing high similarity with bacterial sequences were excluded from the assembly, resulting in the exclusion of 10 contigs with a total length of 6.7 Mbp (Table S2).

### TE annotation

The annotation of transposable elements (TEs) was conducted via both homology and structural search procedures. The initial step involved aligning multiple TE protein databases, including Repbase (version 19.06), REXdb^26^, and TREP databases, against the *S. marianum* reference genome using the fastx32 program with an e-value of 1e-5. Once the alignment was completed, overlapping genomic intervals for each TE and superfamily were merged utilizing Bedtools merge, taking into consideration the insertion strand (-s option). The corresponding nucleotide sequences were subsequently extracted in Fasta format for each superfamily. An ‘all-against-all’ BLASTn search was executed for each superfamily using a minimum e-value of 1e-50. Clustering of different families was performed using the SiLiX program^27^ with a minimum of 80% of identity over 80% of coverage. At this stage of the annotation process, the TE sequences identified represented only the coding regions of the elements, and precise element boundaries were still undefined. Thus, for each paralog within the same family, 10 kbp flanking regions were extracted, and alignment was performed using pblat^28^ to redefine the exact TE boundaries by excising regions that lacked alignment with other paralogs. Once the correct boundaries were identified, multiple sequence alignments were performed using MAFFT^29^, and consensus sequences were generated. This resulted in a total of 408 Class I and 129 Class II elements with consensus sequences. Additionally, we ran LTRharvest^30^ using default parameters except for -xdrop 37 -motif tgca -motifmis 1 -minlenltr 100 -maxlenltr 3000 -mintsd 2. Similar to the strategy described earlier, paralogs were then clustered using SiLiX^27^, and consensus sequences for each family were generated. In total, we identified 563 long terminal repeat (LTR) families. Miniature inverted-repeat transposable elements (MITEs) were identified using MITE-tracker with default parameters. This resulted in the characterization of 443 non-redundant families. TEs identified using in-house strategy, LTR_harvest, and MITE-tracker were merged and redundant families removed, which gave rise at the end to 1239 consensus TE sequences, including 270 Gypsy LTRs, 265 Copia LTRs, 17 LINEs, 49 Mutator, 25 CACTA, 10 Harbinger, 19 Helitrons, and 443 MITEs.

Using the newly characterized 1239 consensus TE sequences, we found that TEs make up 58.01% of the *S. marianum* genome (Table 2). Most of these elements were located in the pericentromeric regions of chromosomes (Fig. 2b, Figure S2). In comparison with other plant genomes, a similar pattern was observed where LTRs emerged as the predominant TE type, contributing 70.46% of total TEs in this species. In Class II, MITEs emerged as the most abundant among terminal inverted repeat transposons (TIRs), accounting for 11.2% of the total genome.

**Table 2:** Summary of transposable elements in *Silybum marianum* cv. Silyking v1.

Code	Class	Order	Superfamily	Total length (bp)	% of genome	% of TE
RLC	Class I	LTR	Copia	165,634,194	23.85	41.15
RLG	Class I	LTR	Gypsy	73,223,946	10.55	18.18
RLX	Class I	LTR	Unknown	44,826,005	6.46	11.13
RIL	Class I	LINEs	Unknown	5,467,739	0.79	1.36
DTC	Class II	TIR	CACTA	12,834,335	1.85	3.19
DTM	Class II	TIR	Mutator	14,770,974	2.13	3.67
DTA	Class II	TIR	hAT	2,264,852	0.33	0.56
DXX	Class II	TIR	MITEs	77,739,850	11.20	19.31
DHH	Class II	Helitron	Helitron	5,879,725	0.85	1.46
Total				402,641,620	58.01%	

### Gene prediction and functional annotation

Protein-coding genes in the assembled genome were predicted using a combination of *ab initio* prediction, transcriptome-based prediction, and protein alignment. Repetitive sequences in the *S. marianum* genome were masked using RepeatMasker v4.0.5. Raw sequences of RNA-seq data were pre-processed (trim, filter, and remove adapters) using Trimmomatic v0.39^20^ with a Q > 20 and 50 bp minimum read length threshold. High-quality reads were then aligned to the assembly using HISAT2 v2.1.0^31^, achieving an average alignment rate of 97.6%. The *ab initio* prediction was carried out with the assistance of BRAKER v1.11^32^, GeneMark-ES/ET v4.48-3.60^33,34^, and AUGUSTUS v3.2.2^35^, utilizing the mapped RNA-seq reads and the assembly with repeat sequences masked. This approach predicted 192,663 genes with a mean exon length of 384 bp. For the transcriptome-based prediction, the high-quality RNA-seq reads were assembled *de novo* using Trinity v2.8.6^36^. The RNA-seq reads were then mapped to the transcriptome assembly and annotated using StringTie v2.0.4^37^. The *de novo* transcriptome assembly and mapped read annotation were aligned against the genome assembly to model complete and partial gene structures using PASA v2.4.1^38^, resulting in the prediction of 101,524 genes with a mean exon length of 321 bp. In addition, the evidence-based gene models were generated using Exonerate v2.2.0^39^ based on the protein sequences of closely related species of *S. marianum*. This approach predicted 52,185 genes with a mean exon length of 250 bp. Lastly, the gene prediction models from *ab initio* prediction, transcriptome-based prediction, and protein alignment were integrated using EvidenceModeler v1.1.1^40^ with different weightings assigned. Subsequently, coding genes lacking start or stop codons or originating from transposable elements were excluded using BLAST v2.9.0, resulting in the prediction of a total of 133,358 gene models.

To investigate the functions of the 133,358 gene models, BLASTp v2.9.0 search was conducted against NCBI plant Refseq DB (7,734,553 sequences), Uniprot DB (565,254 sequences), and TAIR DB (48,356 sequences). In addition, conserved protein domain, gene ontology, and pathway analyses necessary for gene function inference were performed based on Pfam, GO, and KEGG databases using InterProScan v5.38^41^. A gene was considered expressed if the read count within the integrated gene model region in the RNA-seq alignment exceeded zero. The results of the BLASTp, InterProscan, and RNA-seq alignment analyses revealed that 79,862 of the gene models had either associated function or transcript evidence, while 5,779 genes curated as polyproteins were excluded. As a result, 74,083 (55.55%) gene models were selected. Subsequently, a total of 36,163 genes that overlapped with transcriptome-based prediction or protein alignment results were selected. Additionally, 21,266 genes that did not overlap with transcriptome-based prediction or protein alignment but had descriptions at BLASTp and InterProScan results were selected. A total of 3,447 genes with hits to the bacterial genome and 430 genes without hits to the *S. marianum* assembly were excluded. Lastly, a total of 53,552 genes were selected as final gene models with a mean exon length of 289 bp and an average of 3.9 exons per gene (Table 3, Table S3).

**Table 3:** Summary of gene annotation of *Silybum marianum* cv. Silyking v1.

	*S. marianum* cv. Silyking v1
Number of predicted protein-coding genes	53,552
Average gene length (bp)	2,740.1
Average transcript length (bp)	1,135.0
Number of exons	209,677
Average exon length (bp)	289.9
Average exon number per gene	3.9
Number of introns	156,125
Average intron length (bp)	550.5
Completeness BUSCO (%)	96.53
Completeness single-copy BUSCO (%)	92.19
Completeness duplicated BUSCO (%)	4.34

### Comparative genomic analysis

**Fig. 3:**
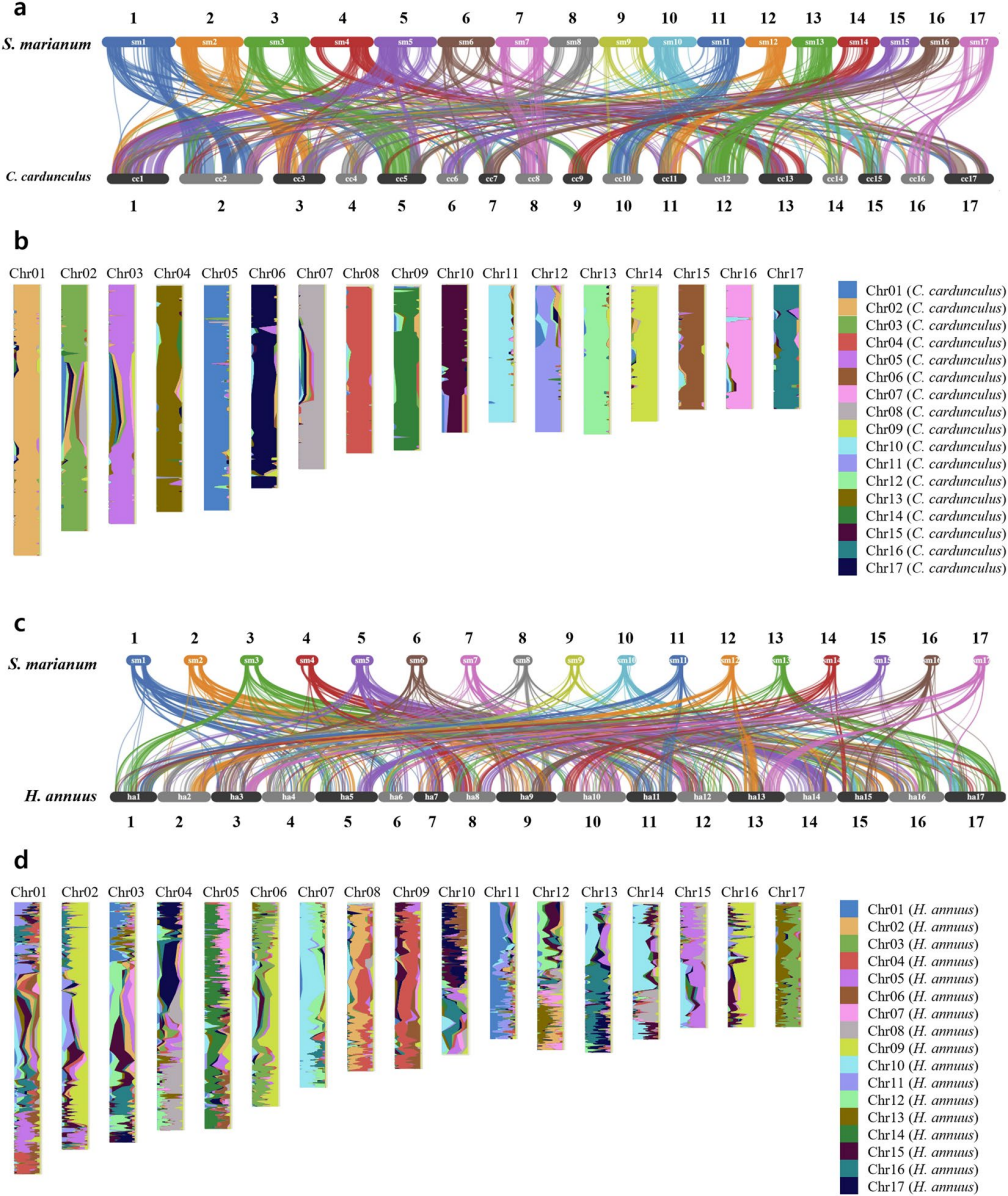
Comparative genome analysis between *Silybum marianum, Cynara cardunculus*, and *Helianthus annuus*. (**a**) Collinearity between *S. marianum* and *C. cardunculus* across 17 chromosomes. (**b**) *S. marianum* chromosomes painted with collinearity regions between *S. marianum* and *C. cardunculus*. (**c**) Collinearity between *S. marianum* and *H. annuus* across 17 chromosomes. (**d**) *S. marianum* chromosomes painted with collinearity regions between *S. marianum* and *H. annuus*.

Collinearity in the *S. marianum* genome was identified through MCScanX^42^ and visualized with Circos v.0.66^43^. Additionally, chromosomal level collinearity was assessed between *S. marianum*, *Cynara cardunculus*, and *Helianthus annuus* using MCScanX^42^ and PanSyn v1.0. The collinearity between *S. marianum* and *C. cardunculus* was highly conserved showing a 1-to-1 relationship of chromosomes (Fig. 3a,b), while that between *S. marianum* and *H. annuus* showed complex and discontiguous patterns (Fig. 3c,d).

**Fig. 4:**
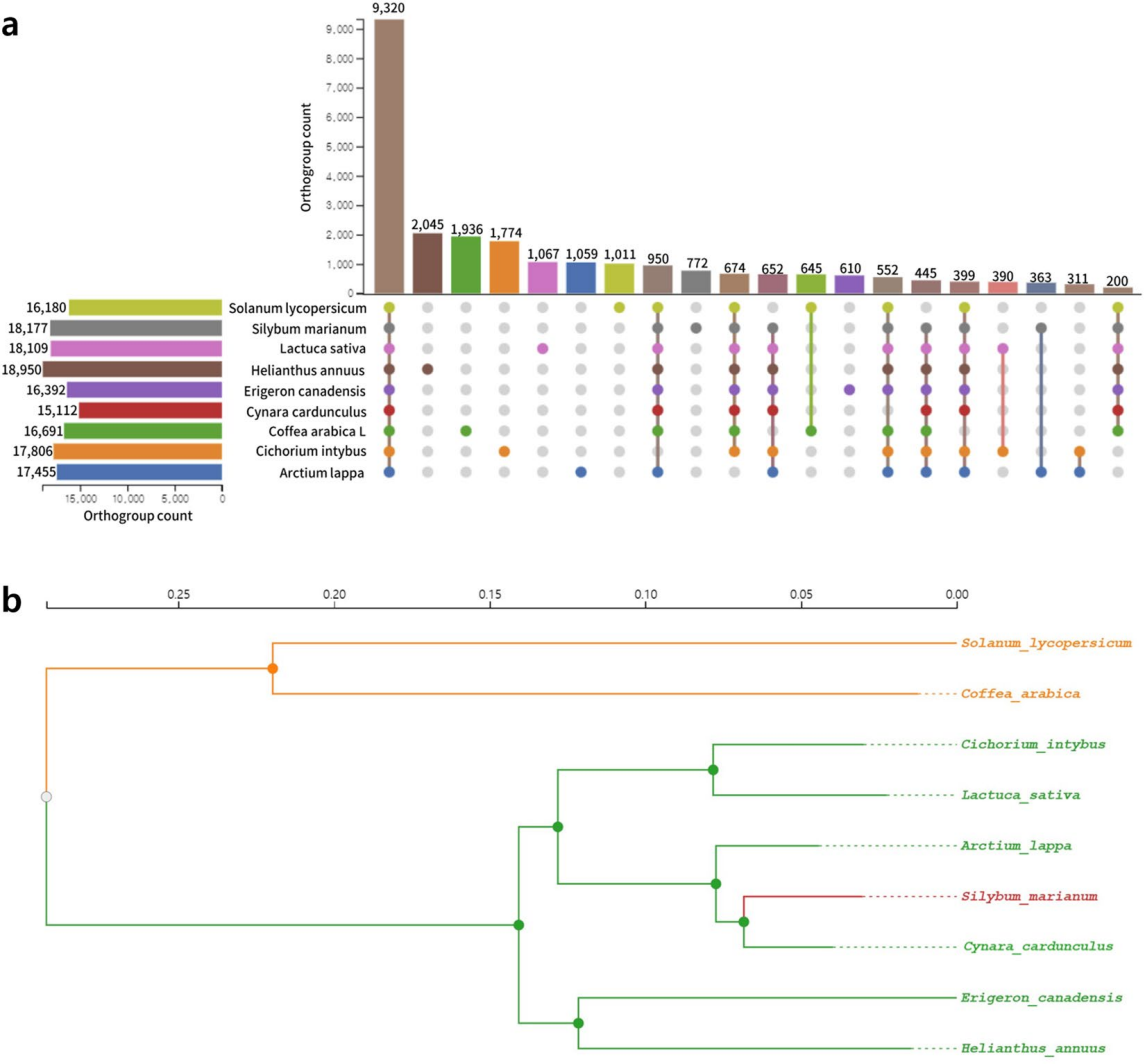
Genome evolution of *Silybum marianum*. (**a**) Top 20 orthogroups between *S. marianum* and eight plant species. See Table S5 for the number of genes per orthogroup. (**b**) Phylogenetic tree of *S. marianum* and eight plant species.

By using OrthoFinder2 v2.3.12^44^ and the protein sequences, orthogroups were identified between *S. marianum* and eight species (Table S4), including *C. cardunculus* (Artichoke, GCA_001531365.2)^45^, *H. annuus* (Common sunflower, GCA_002127325.1)^46^*, Arctium lappa* (Great burdock, GCA_023525745.1)^47^*, Cichorium intybus* (Chicory, GCA_023525715.1)^48^, *Erigeron canadensis* (Horseweed, GCA_010389155.1)^49^, *Lactuca sativa* (Lettuce, GCA_002870075.3)^50^, *Solanum lycopersicum* (Tomato, ITAG4.0), and *Coffea Arabica L*. (Coffee, GCA_003713225.1)^51^. A total of 31,351 orthogroups were identified, comprising 263,955 genes in total (Fig. 4a, Table S5). The phylogenetic tree was constructed using FastTree2^52^ based on the multiple sequence alignments of clustered orthogroups performed using MAFFT v7.3.13^29^ (Fig. 4b).

## Data Records

Chromosome-level genome assembly of *S. marianum* has been deposited at the NCBI GenBank under accession number JAWIMA000000000^53^. Raw data for nanopore sequencing and RNA-seq have been deposited at the NCBI Sequence Read Archive under accession numbers SRR28145636-SRR28145644^54–62^, and are currently available under accession number PRJNA1021369 (https://www.ncbi.nlm.nih.gov/bioproject/PRJNA1021369). The sequences of genome assembly, the annotations of genes and transposable elements, and the list of orthogroups between *S. marianum* and eight species are available at Figshare^63^.

## Technical Validation

### Genome assembly and gene prediction

For the quality assessment of genome assembly, we aligned the sequence reads from both RNA-seq and whole-genome resequencing data into our assembly, showing 97.6% and 99.4% of trimmed reads aligned, respectively (Table 1). Additionally, we checked the completeness of our assembly using BUSCO v4.1.4 with the embryophyte_odb10 database (Figure S3). As a result, the genome assembly from the previous research (ASM154182v1) showed 36.7% of completeness while our assembly from this study showed 99.1% of completeness. Moreover, the continuity of our assembly was evaluated using the LTR Assembly Index (LAI)^64^. The LAI score of our assembly was 17.77, which was higher than that of the Arabidopsis reference genome (TAIR10; LAI = 14.9). Our genome assembly can be considered as ‘reference quality’ with an LAI score ranging from 10 to 20, proposed by Ou *et al*.^64^.

For the validation of gene prediction, we used BUSCO with embryophyte_odb10 and viridiplantae_odb10 databases (Figure S3, Table S6). With the embryophyte database, the predicted *S. marianum* protein-coding genes showed 96.53% of completeness. In the case of the viridiplantae database, predicted *S. marianum* protein-coding genes showed 97.41% of completeness.

### Functional annotation of protein-coding genes

Functional annotation of the predicted genes identified 53,552 genes in *S. marianum* (Table S7). More than 97% (51,994 genes) of predicted genes showed homology with the sequences in the NCBI RefSeq database. Moreover, 50,329 genes (94% of total genes) with functional descriptions in public databases such as NCBI RefSeq, Uniprot, and TAIR were categorized as known proteins. Additionally, 1,853 genes aligned by BLAST but lacking a characterized term and 1,370 genes not aligned by BLAST but showing FPKM > 0.5 in RNA-Seq were categorized as uncharacterized genes.

### Supplementary information

Supplementary Information

## Data Availability

All data processed with publicly available bioinformatics tools or pipelines followed the analysis guidelines provided by those tools. No custom code was used during this study for the curation and/or validation of the dataset.
