# Supplementary material for: Chromosome-level genome assembly of milk thistle (Silybum marianum (L.) Gaertn.)
Source: Sci Data. 2024 Apr 5;11:342. doi: 10.1038/s41597-024-03178-3 (PMC10997770; doi:10.1038/s41597-024-03178-3)
Supplement: Supplementary file 1 — Supplementary Information [file 41597_2024_3178_MOESM1_ESM.pdf]

Supplementary Information

Title
Chromosome-level genome assembly of milk thistle (*Silybum marianum* (L.) Gaertn.)

Authors
Kyung Do Kim[1, *], Jeehyoung Shim[2], Ji-Hun Hwang[1], Daegwan Kim[3], Moaine El Baidouri[4,5], Soyeon Park[1], Jiyong Song[1,3], Yeisoo Yu[3], Keunpyo Lee[6], Byoung-Ohg Ahn[7], Su Young Hong[7,*], Joong Hyoun Chin[8,9, *]

Affiliations
1. Department of Biosciences and Bioinformatics, Myongji University, Yongin 17058, Korea
2. EL&I Co., Ltd., Hwaseong 18278, Korea
3. Department of Research and Development, DNACARE Co. Ltd., Seoul 06126, Korea
4. Laboratoire Génome et Développement des Plantes, Center National de la Recherche Scientifique (CNRS), Perpignan, France
5. Laboratoire Génome et Développement des Plantes, University of Perpignan Via Domitia, Perpignan, France
6. International Technology Cooperation Center, Technology Cooperation Bureau, Rural Development Administration, Jeonju 54875, Korea
7. Genomics Division, Department of Agricultural Biotechnology, National Institute of Agricultural Science, Rural Development Administration, Jeonju 54874, Korea
8. Food Crops Molecular Breeding Laboratory, Department of Integrative Biological Sciences and Industry, Sejong University, Seoul 05006, Korea
9. Convergence Research Center for Natural Products, Sejong University, Seoul 05006, Korea

*These authors have contributed equally to this work
Corresponding author(s): Kyung Do Kim (kyungdokim@mju.ac.kr), Su Young Hong (suyoung@korea.kr), Joong Hyoun Chin (jhchin@sejong.ac.kr)

Table of Contents

Figure S1. Genome survey of *Silybum marianum* using k-mer analysis.
Figure S2. Heatmap of TE density across 17 chromosomes.
Figure S3. BUSCO assessment of *Silybum marianum*.
Table S1. Summary of Pore-C scaffolding.
Table S2. Unplaced contigs showing high similarity with bacterial sequences.
Table S3. Chromosome-level summary of genome assembly and gene annotation of *Silybum marianum* cv. Silyking v1.
Table S4. Summary of nine plant species for comparative genomic analysis in this study.
Table S5. Orthologous genes from top 20 orthogroups determined across nine plant species.
Table S6. BUSCO assessment of gene annotation of *Silybum marianum* cv. Silyking v1.
Table S7. Summary of functional gene annotation of *Silybum marianum* cv. Silyking v1.

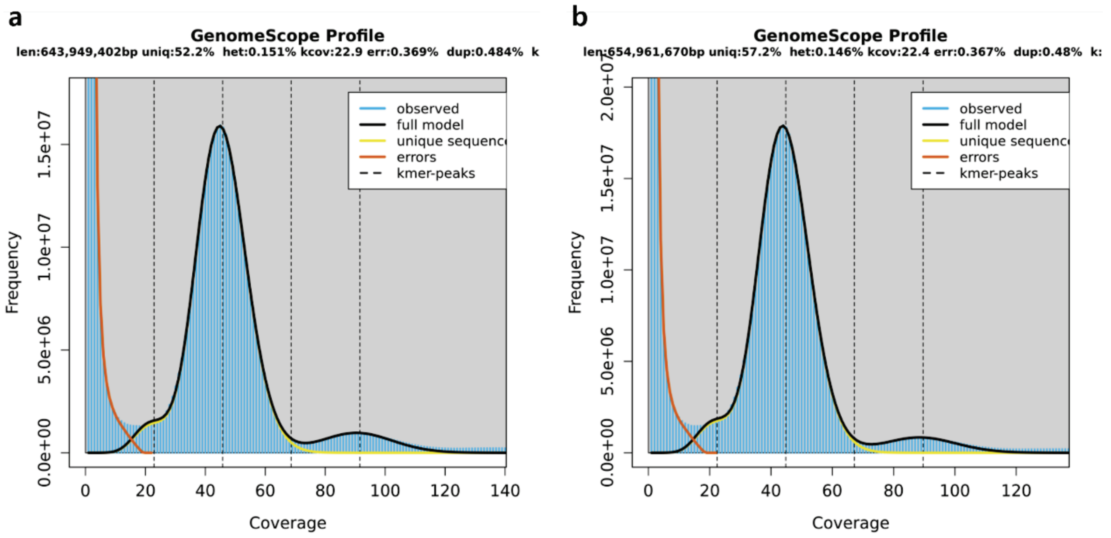

Figure S1. Genome survey of *Silybum marianum* using k-mer analysis. (a) GenomeScope profile using 19-mer. The genome size was estimated as 643 Mb with 0.151% heterozygosity. (b) GenomeScope profile using 21-mer. The genome size was estimated as 654 Mb with 0.146% heterozygosity.

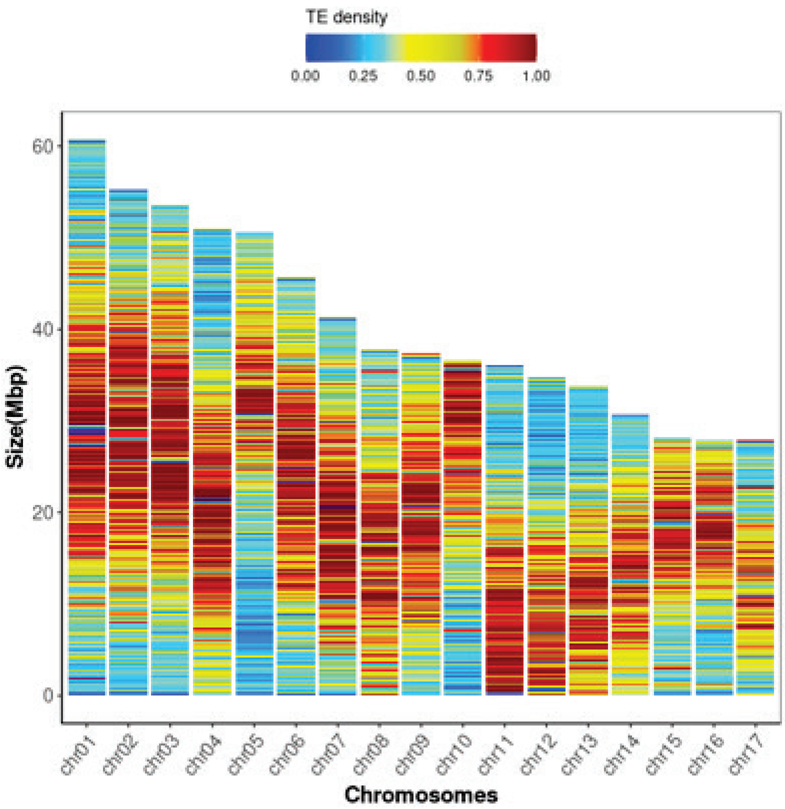

Figure S2. Heatmap of TE density across 17 chromosomes. The color intensity represents the level of TE density. Red color shows the high TE density while blue color shows the low TE density.

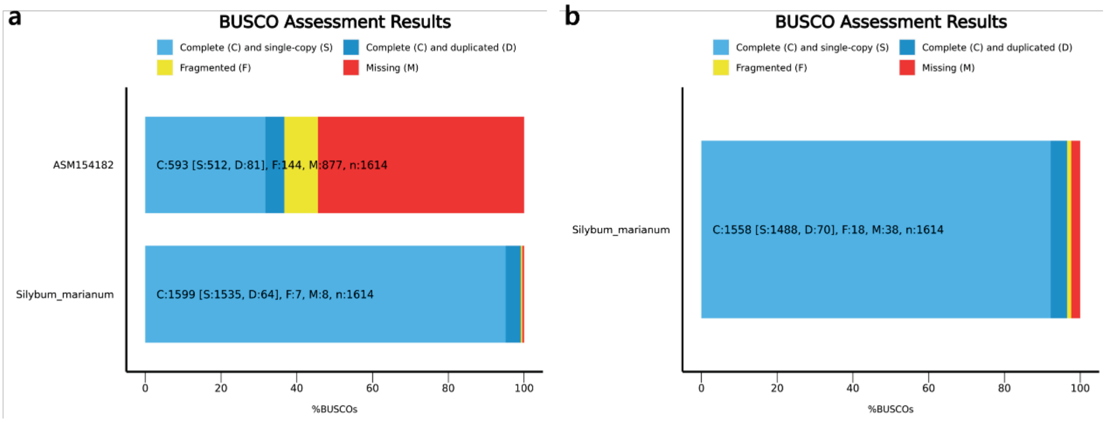

Figure S3. BUSCO assessment of *Silybum marianum*. A core gene set of embryophytes_odb10 was used for the assessment. (a) BUSCO completeness assessment for genome assemblies of *S. marianum*. Top: ASM154182v1, Bottom: cv. Silyking v1. (b) BUSCO completeness assessment for gene annotation of *S. marianum* cv. Silyking v1.

Table S1. Summary of Pore-C scaffolding.

Scaffold name	Linkage group	Contig ordered in Hi-C scaffold	No. of contigs	Length (bp)
HiC_scaffold_1	LG3	T_ctg000210, ctg000260, ctg000620_T	3	53,687,286
HiC_scaffold_2	LG12	T_ctg000690_T	1	60,724,247
HiC_scaffold_3	-	ctg000630R*, ctg000200_T	2	41,395,897
HiC_scaffold_4	LG1	T_ctg000310R, ctg000150R_T	2	45,671,120
HiC_scaffold_5	-	T_ctg000120, ctg000640	2	37,349,463
HiC_scaffold_6	LG8	ctg000380	1	34,728,500
HiC_scaffold_7	LG7	T_ctg000360, ctg000370, ctg000430, ctg000600_T	4	36,652,346
HiC_scaffold_8	-	ctg000350_T	1	36,062,500
HiC_scaffold_9	LG10	T_ctg000660R_T	1	28,119,000
HiC_scaffold_10	LG5	T_ctg000320R_T	1	27,846,507
HiC_scaffold_11	-	T_ctg000340_T	1	33,800,049
HiC_scaffold_12	LG11	T_ctg000670R_T	1	30,639,307
HiC_scaffold_13	LG2	T_ctg000170R_T	1	27,916,000
HiC_scaffold_14	-	T_ctg000160R, ctg000010, ctg000530R, ctg000590R, ctg000610R, ctg000110, ctg000100R, ctg000140_T	8	50,951,241
HiC_scaffold_15	LG4	T_ctg000220_T	1	50,660,146
HiC_scaffold_16	-	ctg000180R, ctg000040, ctg000300_T	3	37,813,352
HiC_scaffold_17	LG6+ LG9	T_ctg000650R, ctg000330_T	2	55,284,169
17 scaffolds	12 LGs	-	35	689,301,130

Table S2. Unplaced contigs showing high similarity with bacterial sequences.

Contig ID	Scaffold	Contig length (bp)	Contaminant
ctg000250	Hi-C_scaffold_20	4,097,170	*Pseudomonas parafulva*
ctg000420	Hi-C_scaffold_21	715,726	*Pseudomonas parafulva*
ctg000440	Hi-C_scaffold_115	372,396	*Pseudomonas parafulva*
ctg000680	Hi-C_scaffold_118	370,928	*Rippkaea orientalis*
ctg000390	Hi-C_scaffold_120	366,884	*Rippkaea orientalis*
ctg000090	Hi-C_scaffold_127	318,409	*Magnetospira*
ctg000030	Hi-C_scaffold_22, 139, 140	224,109	*Lactobacillus delbrueckii*
ctg000290	Hi-C_scaffold_27	135,776	*Acinetobacter baumannii*
ctg000280	Hi-C_scaffold_29	86,836	*Acinetobacter baumannii*
ctg000270	Hi-C_scaffold_30	59,754	*Acinetobacter baumannii*
10 contigs	12 scaffolds	6,747,988	

Table S3. Chromosome-level summary of genome assembly and gene annotation of *Silybum marianum* cv. Silyking v1.

Chromosome	Length (bp)	No. of genes	Gene density (kbp / gene)
Chr01	60,724,247	4,688	13.0
Chr02	55,284,169	4,224	13.1
Chr03	53,687,286	3,861	13.9
Chr04	50,951,241	4,180	12.2
Chr05	50,660,146	4,559	11.1
Chr06	45,671,120	3,453	13.2
Chr07	41,395,897	3,166	13.1
Chr08	37,813,352	2,739	13.8
Chr09	37,349,463	2,583	14.5
Chr10	36,652,346	2,993	12.2
Chr11	36,062,500	3,013	12.0
Chr12	34,728,500	2,861	12.1
Chr13	33,800,049	2,623	12.9
Chr14	30,639,307	2,201	13.9
Chr15	28,119,000	2,109	13.3
Chr16	27,916,000	2,125	13.1
Chr17	27,846,507	2,076	13.4
Unplaced Contigs	5,065,881	98	51.6

Table S4. Summary of nine plant species for comparative genomic analysis in this study.

Scientific name	Common name	Family	Number of chromosomes	Genome size (Mb)	Number of genes	Genome version	Reference
Silybum marianum	Milk thistle	*Asteraceae*	17	689	53,552	Smar.v1	This study
Cynara cardunculus var. *scolymus*	Artichoke	*Asteraceae*	17	725	26,498	CcrdV1.1	Acquadro et al. (2017)
Arctium lappa	Great burdock	*Asteraceae*	18	1,727	46,935	ASM2352574v1	Wang et al. (2022)
Cichorium intybus	Chicory	*Asteraceae*	9	1,279	53,946	ASM2352571v1	Salvagnin et al. (2023)
Lactuca sativa	Lettuce	*Asteraceae*	10	2,400	38,910	Lsat_Salinas_v8	Reyes-Chin-Wo et al. (2017)
Erigeron canadensis	Horseweed	*Asteraceae*	9	426	33,472	C_canadensis_v1	Laforest et al. (2020)
Helianthus annuus	Common sunflower	*Asteraceae*	17	3,010	83,587	HanXRQr2.0-SUNRISE	Badouin et al. (2017)
Solanum lycopersicum	Tomato	*Solanaceae*	13	783	34,075	ITAG4.0	Hosmani et al. (2019)
Coffea Arabica L.	Coffee	*Rubiaceae*	11	1,094	56,902	Cara_1.0	Scalabrin et al. (2020)

Table S5. Orthologous genes from top 20 orthogroups determined across nine plant species.

Orthogroups	Number of orthologous genes									
	A. lappa	*C. intybus*	*C. arabica L*	*C. cardunculus*	*E. canadensis*	*H. annuus*	*L. sativa*	*S. marianum*	*S. lycopersicum*	Total
9,320	15,263	15,909	19,760	14,571	17,950	20,236	16,775	16,051	14,318	150,833
2,045	-	-	-	-	-	7,745	-	-	-	7,745
1,936	-	-	9,450	-	-	-	-	-	-	9,450
1,774	-	6,623	-	-	-	-	-	-	-	6,623
1,067	-	-	-	-	-	-	4,878	-	-	4,878
1,059	5,043	-	-	-	-	-	-	-	-	5,043
1,011	-	-	-	-	-	-	-	-	4,658	4,658
950	1,168	-	1,900	1,175	1,452	1,584	1,256	1,220	1,363	11,118
772	-	-	-	-	-	-	-	10,779	-	10,779
674	-	878	1,334	811	1,072	1,236	941	848	932	8,052
652	1,086	1,152	-	1,021	1,360	1,724	1,507	1,366	-	9,216
645	-	-	2,134	-	-	-	-	-	1,187	3,321
610	-	-	-	-	3,234	-	-	-	-	3,234
552	695	764	1,193	-	863	966	798	707	784	6,770
445	1,037	716	966	741	921	1,038	778	935	-	7,132
399	801	780	-	638	838	1,081	964	823	576	6,501
390	-	952	-	-	-	-	1,055	-	-	2,007
363	1,744	-	-	-	-	-	-	1,642	-	3,386
311	627	652	-	-	-	-	-	-	-	1,279
200	-	-	385	218	262	313	246	253	253	1,930
Total	27,464	28,426	37,122	19,175	27,952	35,923	29,198	34,624	24,071	263,955

Table S6. BUSCO assessment of gene annotation of *Silybum marianum* cv. Silyking v1.

**Ortholog Database**	**Viridiplantae**		**Embryophyta**	
**Parameter**	**Count**	**Rate (%)**	**Count**	**Rate (%)**
Complete BUSCO (C)	414	97.41	1,558	96.53
Complete and single-copy BUSCO (S)	404	95.06	1,488	92.19
Complete and duplicated BUSCO (D)	10	2.35	70	4.34
Fragmented BUSCO (F)	5	1.18	18	1.12
Missing BUSCO (M)	6	1.41	38	2.35
**Total BUSCO groups**	425	-	1,614	-

Table S7. Summary of functional gene annotation of *Silybum marianum* cv. Silyking v1.

**Classified gene function**		**Gene model**	
		**Gene count**	**Percent**
Known protein		50,329	93.98
Uncharacterized protein	BLASTP with unknown, uncharacterized term)	1,853	3.46
	Expressed protein (RNA-Seq FPKM>0.5)	1,370	2.56
**Total gene model**		53,552	-
